# Uterine Tampon

**Published:** 1893-03

**Authors:** 


UTERINE TAMPON.—Auvard, before the First International Congress for Gynecology and Obstetrics (*Munchener medicinische Wochenschrift*, 39 Jahrgang, No. 42), advocated the tamponing of the uterus with iodoform gauze in all forms of hemorrhage from that organ. In twelve cases of hemorrhage after labor so treated two died—one from tuberculosis two months post partum, and one from septicemia on the tenth day. In twelve cases of hemorrhage following abortion, part before and part after the perfect ejection of the conceived products so treated, Auvard had excellent results, as in one case of bleeding after expulsion of the placenta. Also in bleeding after curettage in uterine carcinoma and of the uterine cavity and after extirpation of uterine myomas through the vagina.—*Ex.*

